# Supplementary material for: Compartmentalized mitochondrial ferroptosis converges with optineurin-mediated mitophagy to impact airway epithelial cell phenotypes and asthma outcomes
Source: Nat Commun. 2024 Jul 10;15:5818. doi: 10.1038/s41467-024-50222-2 (PMC11237105; doi:10.1038/s41467-024-50222-2)
Supplement: Supplementary file 1 — Supplementary Information [file 41467_2024_50222_MOESM1_ESM.pdf]

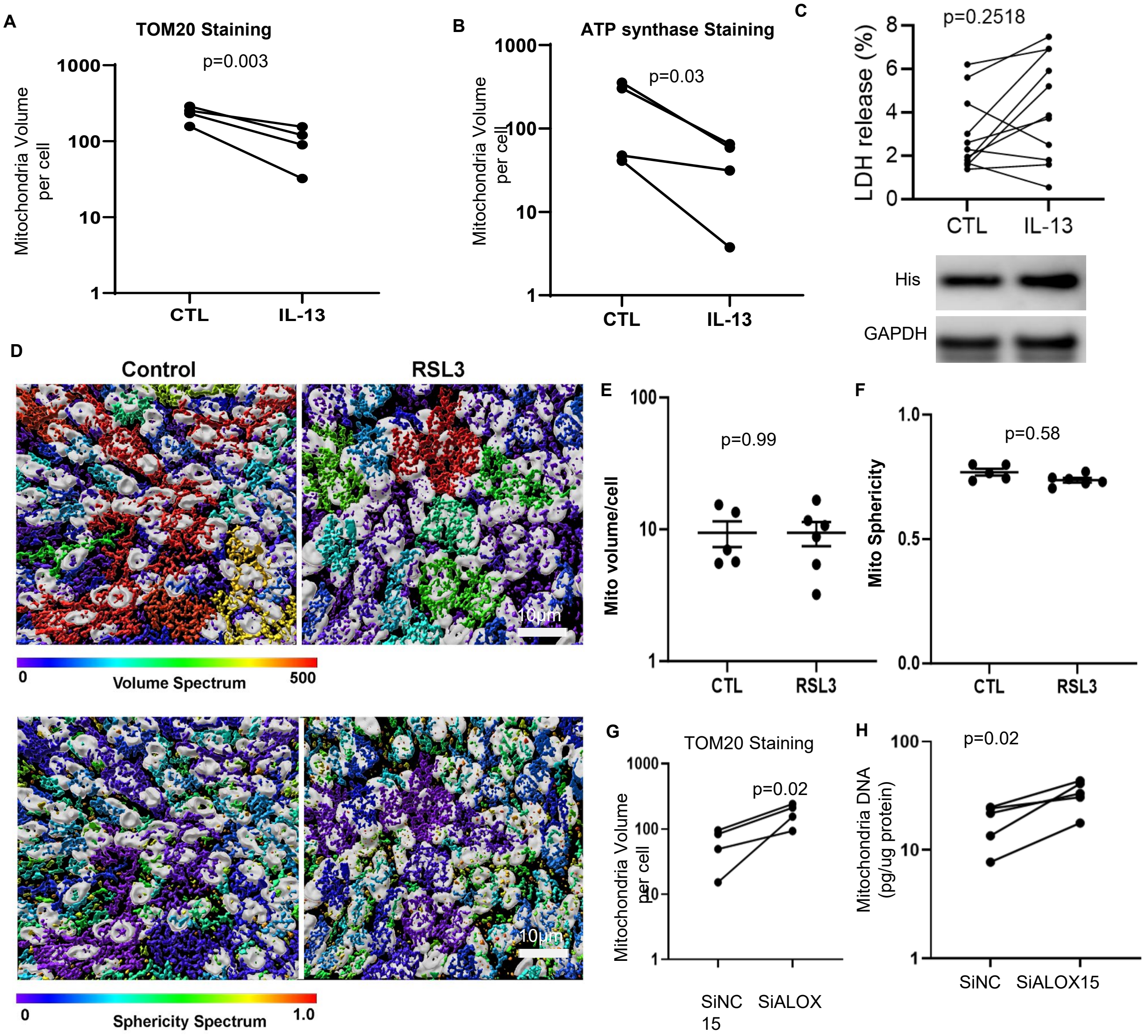

Supplementary Fig. 1. IL-13 induces mitochondria loss via 15LO1-dependent compartmentalized ferroptosis. A) IL-13 decreases mitochondrial volume in HAECs using image analysis of IF/CF TOM20 and ***B*)** ATPsynthase (n=4 biologic replicates, paired t-tests) **C*)*** IL-13 (10ng/ml, 7 days) does not induce cell death measured by LDH release (n=10 biologic replicates, paired t-test) or by nuclear DNA loss analysis (WB of Histidine) (n=4 biologic replicates). ***D-F)*** RSL3 (2μM, 2 hours) treatment of control cells (no IL-13, with low 15LO1 expression) did not impact mitochondria number, size and fragmentation/sphericity (DMSO as vehicle control (CTL) by paired t-test. Cells were fixed and stained with TOM20 for **m**itochondria volume assessment using surface rendering of confocal z-stacks (Imaris, Bitplane) and pseudo-colored as indicated by "*volume spectrum*", and the size and shape of the mitochondria as reflected by the sphericity (n=5 biologic replicates, unpaired t-test) **G)** siALOX15 transfection restored IL-13-induced mitochondria loss by paired t-test (n=5 biologic replicates). **H)** Dicer siALOX15 knockdown reverses IL-13-induced mitochondrial DNA loss by paired t-test (n=5 biologic replicates). Data are normalized to total cell lysate protein. Source data provided as Source Data file.

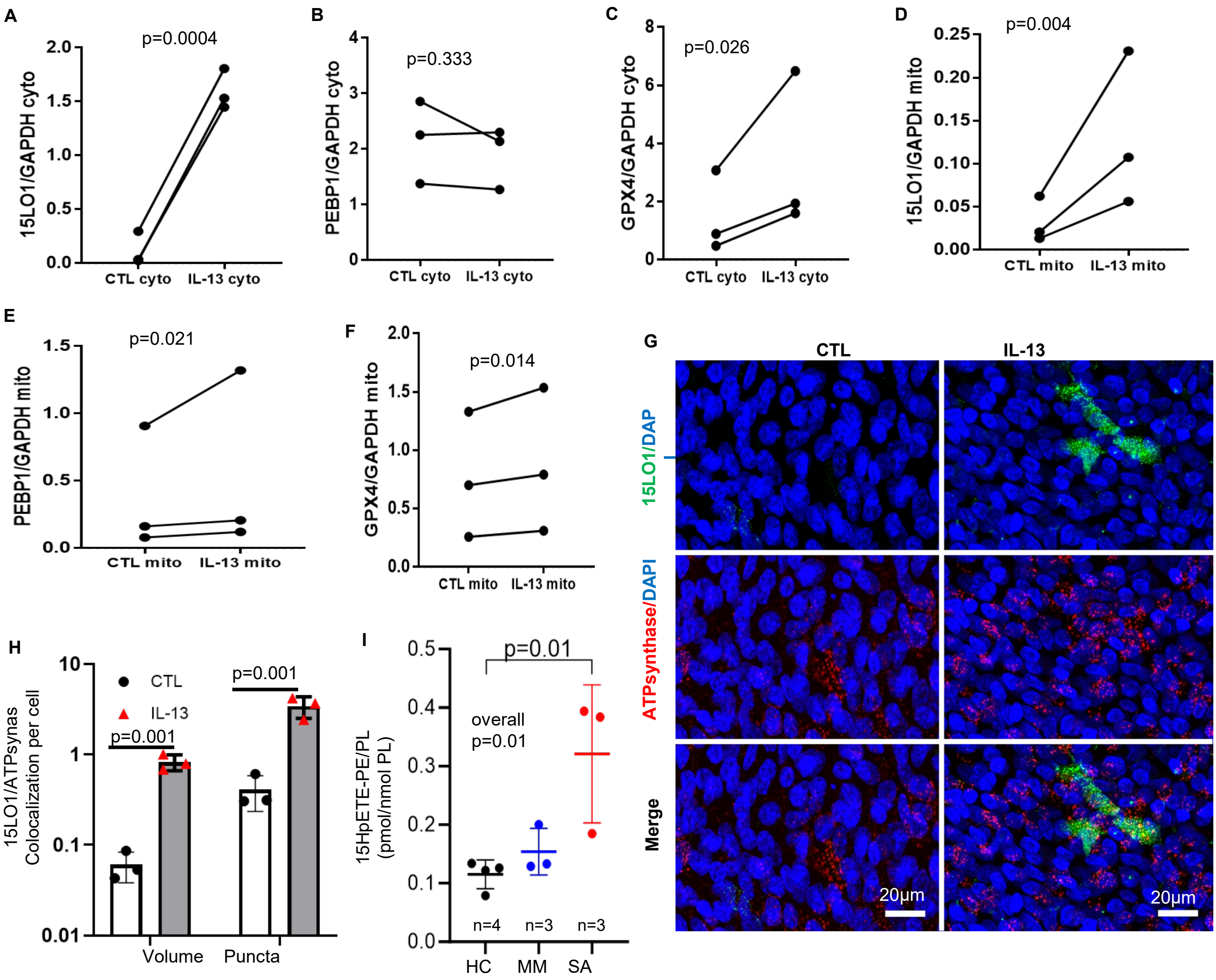

Supplementary Fig. 2. IL-13 increases 15LO1/ATPsynthase colocalization in HAECs *in vitro*. A-F) IL-13 induces ferroptosis-associated 15LO1 protein expression in the mitochondrial fractions by densitometry analysis and paired t-tests with n=3 biologic replicates. **G)** IL-13 increases 15LO1/ATPsynthase colocalization in HAECs by IF/CF (Red: ATPsynthase; Green: 15LO1; Blue: DAPI; Yellow: colocalization). Representative images from n=3 biologic replicates. **H)** IL-13 increases 15LO1/ATPsynthase colocalization volume and puncta in HAECs using image analysis of IF/CF by paired t-testing, n=3 biologic replicates). Data are normalized to the numbers of cells counted and presented as mean ± s.d. **I)** 15HpETE-PE levels are higher in freshly brushed HAECs from severe asthmatic (SA) as compared to mild-moderate (MM) asthmatic and healthy control (HC) participants indexing to total ETE-PE (by LC/MS analysis). One way ANOVA test, with unpaired t-test for intergroup comparison based on biological samples from n of 3-4 individual donor in each group. Source data are provided as Source Data file.

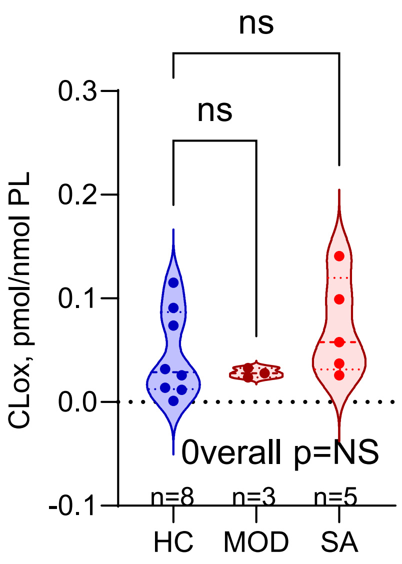

Supplementary Fig. 3. LC/MS analysis showing low amounts of CLox in primary HAECs ex vivo, without significant differences between healthy control (HC) and moderate (MOD) and severe asthmatic (SA) samples. One way ANOVA test, with unpaired t-test for intergroup comparison based on biological samples from n=3-8 individual donor in each group. Source data are provided as Source Data file.

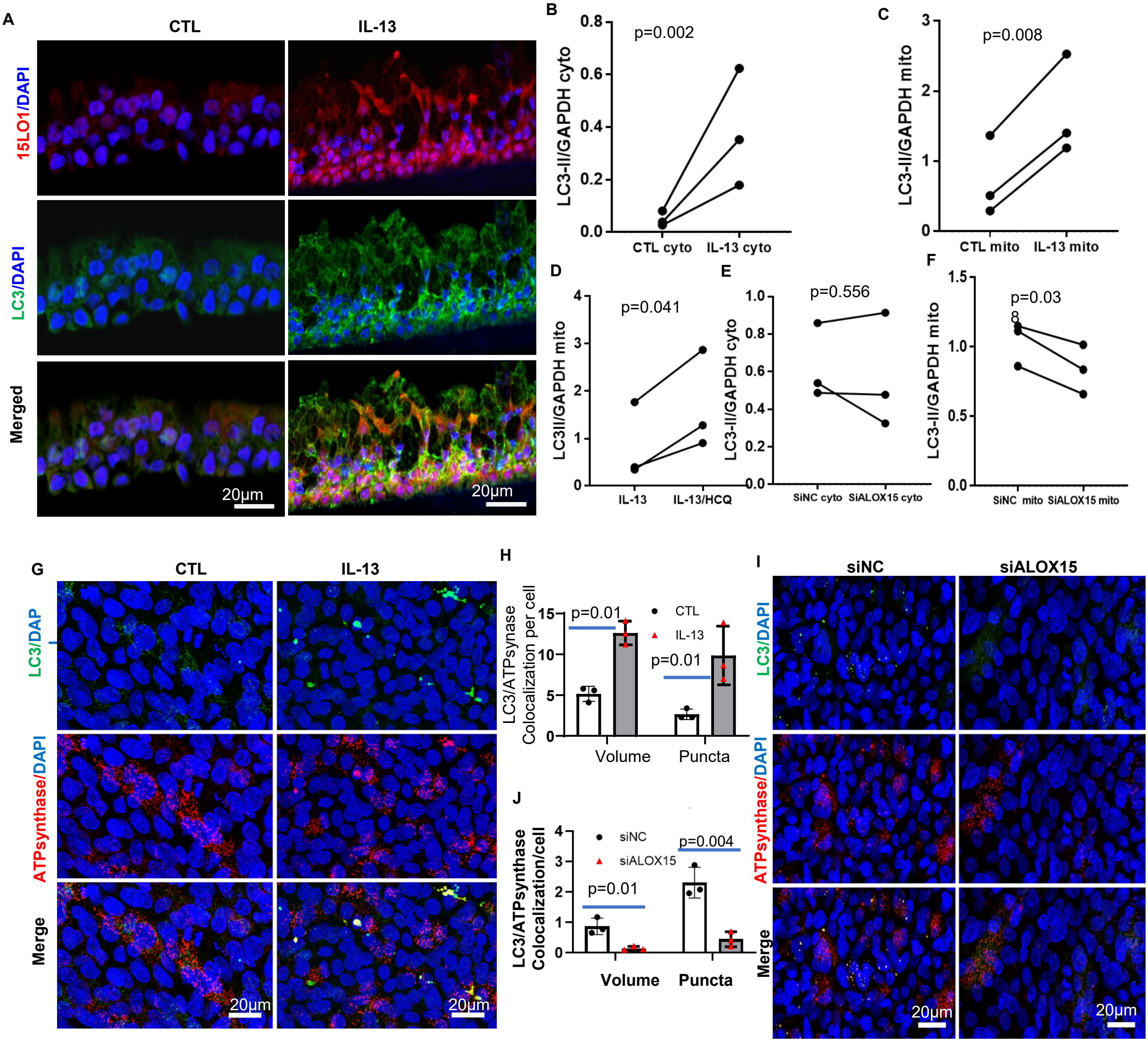

Supplementary Fig. 4. Compartmentalized ferroptosis increases activated mitochondrial LC3 and mitophagy. A) IL-13 increased 15LO1/LC3 expression and colocalization in HAECs, as compared to media control *(CTL). (IF/CF* Red: 15LO1; Green: LC3; Blue: DAPI; Yellow: merge. Representative images from n=3 biologic replicates. IL-13 increases LC3-II in **B)** cytosolic (cyto) fractions and **C)** in mitochondrial (mito) fractions and **D)** enhanced by hydroxychloroquine (HCQ) treatment by densitometry analysis and paired t-tests. Impact of siALOX15 KD under IL-13 conditions on **E)** cytosolic and **F)** mitochondria LC3-II (GAPDH as the loading control and paired t-test on n of 3 biologic replicates. **G)** LC3/ATPsynthase colocalization under IL-13 condition in HAECs (n=3 biologic replicates). (IF/CF Red: ATPsynthase; Green: LC3; Blue: DAPI; Yellow: colocalization in HAECs **H)** quantification by volume and number of puncta using image analysis and paired t-testing on n of 3 biologic replicates. **I)** siALOX transfection reduced IL-13-induced LC3/ATPsynthase colocalization (n=3 biologic replicates). Red: ATPsynthase; Green: LC3; Blue: DAPI; Yellow: colocalization. **J)** quantification by volume and number of puncta using image analysis and paired t-test on n of 3 biologic replicates. Source data provided as Source Data file.

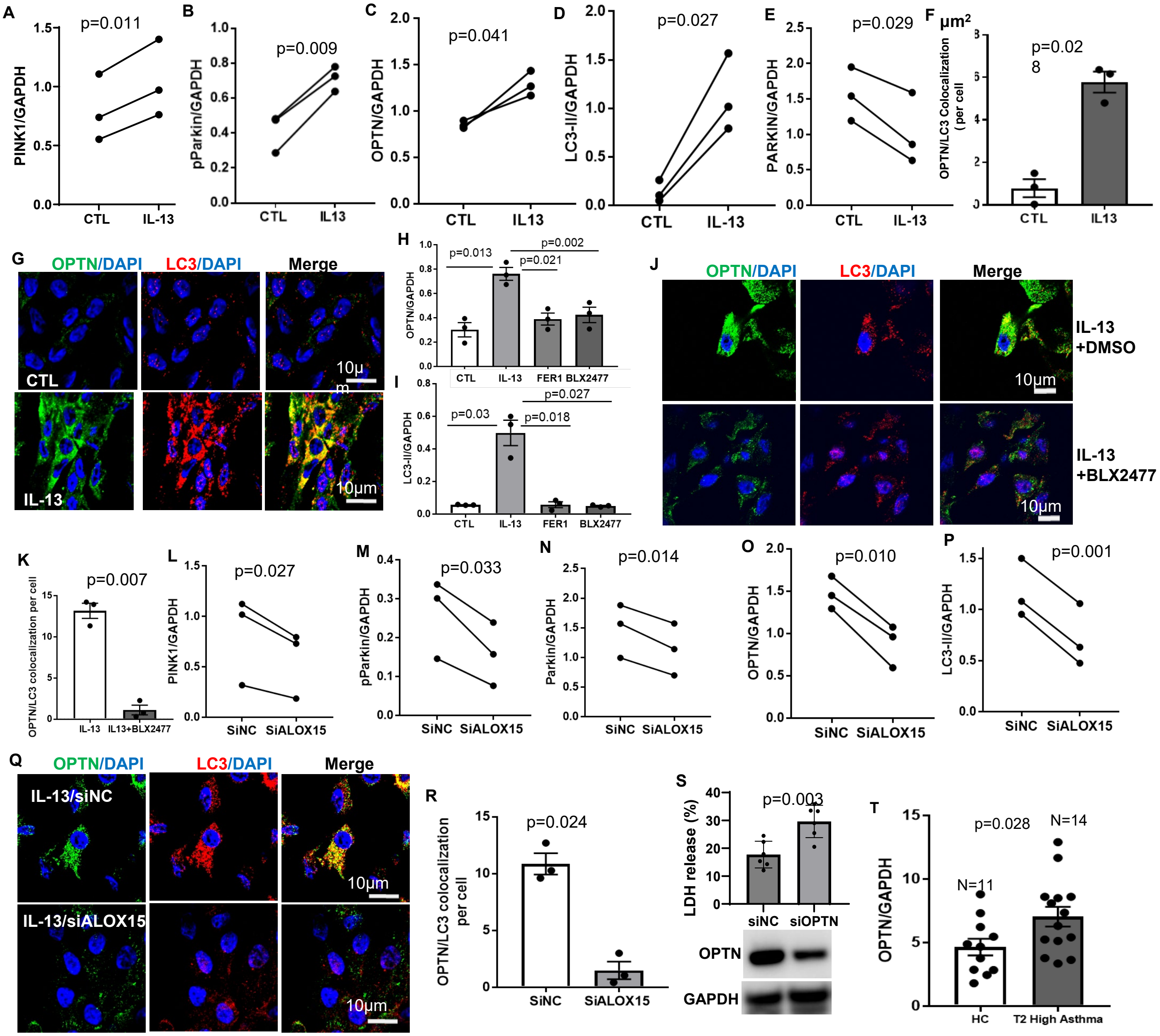

Supplementary Fig. 5. 15LO1-dependent ferroptotic processes activate PINK/Parkin/Optineurin mitophagy. A-D) PINK1/pParkin/Optineurin (OPTN)/LC3-II increase while **E)** Parkin decreases under IL13 conditions, by densitometric analysis and paired t-testing on n=3 biologic replicates. **F)** OPTN co-localization with LC3 under IL-13 conditions using image analysis and paired t-testing on n-=3 biologic replicates(IF/CF) **G)** OPTN colocalizes with LC3 under IL-13 conditions (IF/CF). Green: OPTN; Red: LC3; Blue: DAPI; Yellow: merge. FER-1 and/or BLX2477 suppress IL-13-induced **H)** OPTN and **I)** LC3-II, with GAPDH as the loading control (densitometry with paired t-testing on n=3 biologic replicates) **J)** 15LO1 inhibition with BXL2477(2μM, overnight) lowers OPTN/LC3 colocalization in HAECs under IL-13 conditions. Green: OPTN; Red: LC3; Blue: DAPI; Yellow: merge. Representative image from on n=3 biologic replicates. **K)** Quantification of BLX2477 effects on OPTN/LC3 colocalization by image analysis and paired t-testing (n=3 biologic replicates) (IF/CF). **L-P)** 15LO1 KD by siALOX05 lowers IL-13-induced PINK1/pParkin/OPTN /LC3-II by densitometry with paired t-testing (n=3 biologic replicates). **Q)** 15LO1 KD/siALOX15 lowers OPTN/LC3 colocalization in HAECs under IL-13 conditions Green: OPTN; Red: LC3; Blue: DAPI; Yellow: merge **R)** image analysis of colocalizations with paired testing on n of 3 biologic replicates. S**)** OPTN KD using siOPTN (see WB below) increases LDH release under IL-13 conditions by paired t-testing on n=3 biologic replicates. **T)** OPTN expression (indexed to GAPDH) by WB is higher Type-2 (T2)-high asthmatic participants [fraction exhaled NO (FeNO)>25] as compared to heatlhty controls (HC) by unpaired t-testing on n =11-14 biologic samples from different individual donors. Source data provided as Source Data file.

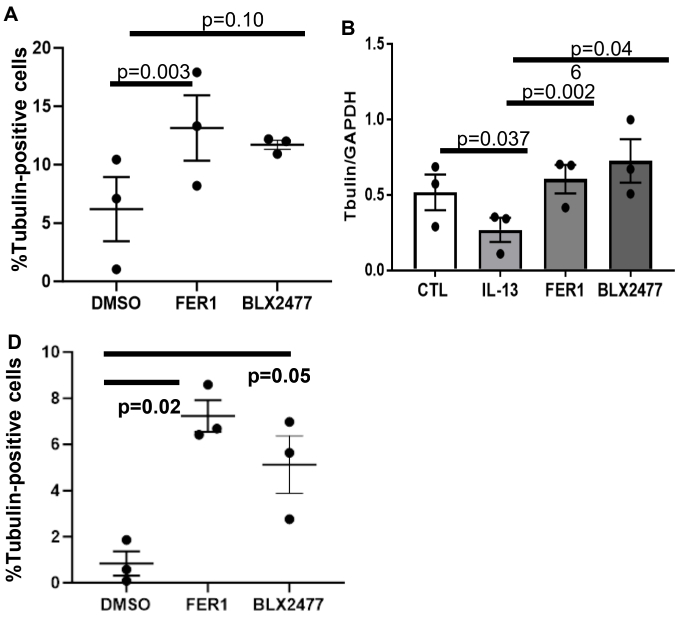

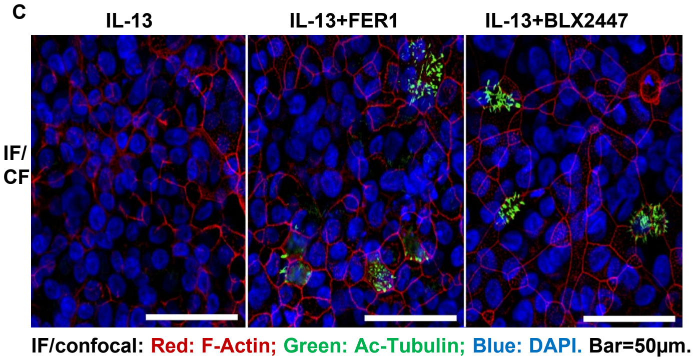

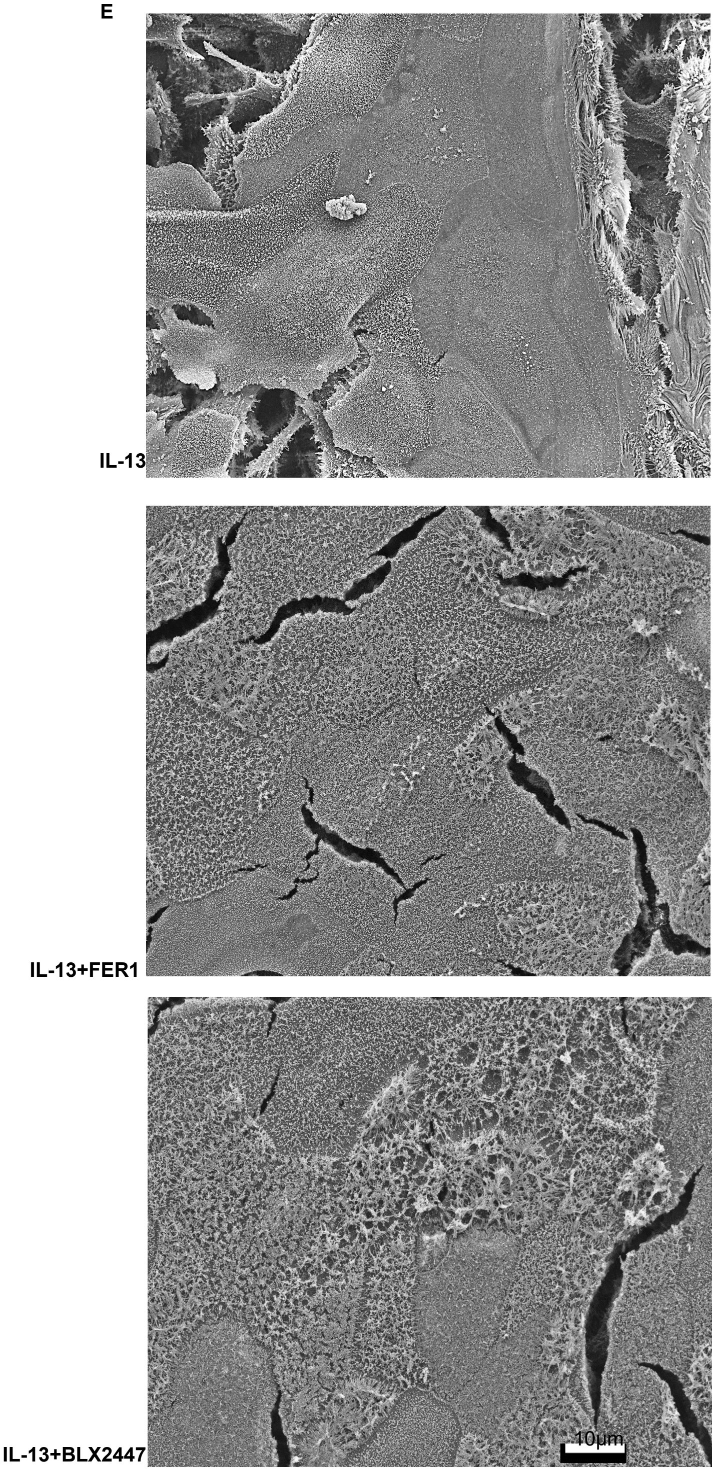

Supplementary Fig. 6. Functional implications of 15LO1-dependent ferroptosis. A) Ferristatin-1 (FER-1) (1μM, 5 days) and/or BLX2477(2μM,5 days) increase ciliated cell numbers under IL-13 for 5 days (after 9 days in ALI) using image analysis and paired t-testing (n of 3 biologic replicates). **B)** Similar treatment with FER-1 (1μM, 5 days) and/or BLX2477(2μM,5 days) increase tubulin (TUB1A) expression under IL-13 conditions by paired t-testing (n=3 biologic replicates)). **C)** IL-13(10ng/ml, 7 days) at ALI day 0 almost completely inhibits ciliated cell development, while treatment with FER-1 (1μM, 7 days) and/or BLX2477(2μM, 7 days) increases ciliated cells (IF/CF). Representative image from on n=3 biologic replicates. **D)** Image quantification demonstrates near complete lack of cilia under IL-13 conditions at Day 7 of ALI, with increases in ciliated cell percentages following treatment with FER-1 and BLX2477 (paired t-testing with n=3 biologic replicates), confirmed by **E)** SEM (Representative image from on n=3 biologic replicates). Scale bar: 10μm. from on n=3 biologic replicates Source data provided as Source Data file.

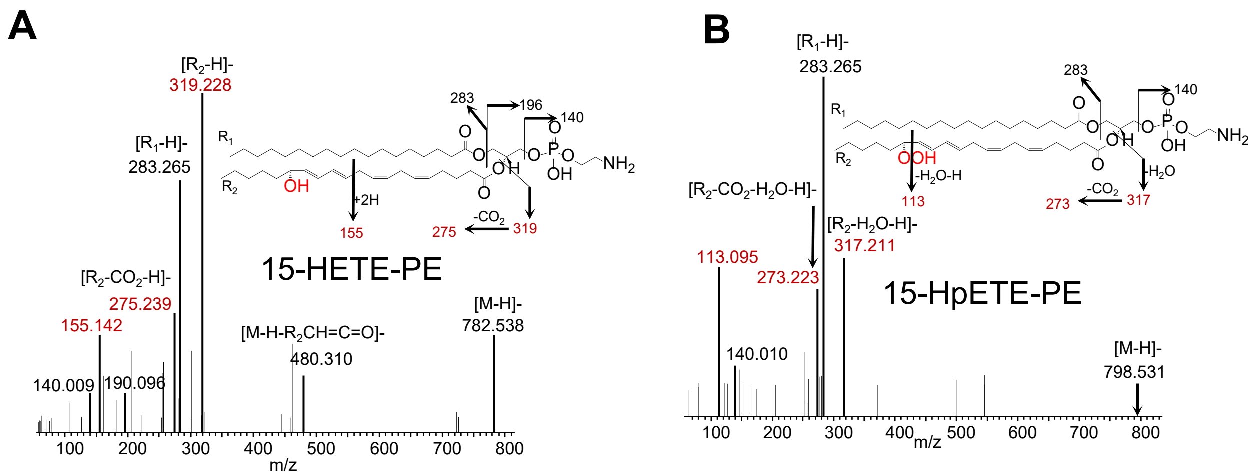

Supplementary Fig. 7. LC-MS/MS analysis of oxygenated phospholipids in HAECs. A) MS/MS spectrum of PE molecular ion with m/z 782.538 at RT = 80.67 min and the structure of oxidized PE species containing hydroperoxy-arachidonic acid with assigned fragments (inserts). The fragment with m/z 283.265 [R1-H] corresponds to stearic acid (C18:0) that is localized in the sn-1 position of PE. Fragment with m/z 319.228 [R2-H]- corresponding to C20:4-OH and ion with m/z 275.239 formed from hydroxy-arachidonic acid with loss of CO2 [R2-CO2-H]- were observed in the MS2 spectrum. The fragment with m/z 480.310 [M-H-R2CH=C=O]- was generated with loss of C20:4-OOH as ketene. Fragment m/z 155.142 is produced after C10-C11 bond cleavage and addition of two hydrogens. Fragments attributed to polar head of PE with m/z 140.009 and 196.096 were also formed during MS2 fragmentation. Based on fragmentation pattern the species with m/z 782.538 was identified as 15-HETE-PE. **B)** MS/MS spectrum of PE molecular ion with m/z 798.531 at RT = 78.65 min and the structures of oxidized PE species containing hydroperoxy-arachidonic acid with assigned fragments (inserts). The fragment with m/z 283.265 [R1-H] corresponds to stearic acid (C18:0) that is localized in the sn-1 position of PE. Fragment with m/z 317.211 [R2-H]- is formed after dehydration of hydroperoxy-arachidonic acid and ion with m/z 273.223 is formed from dehydrated ion with loss of CO2 [R2-H2O-CO2-H]-. Fragment m/z 113.095 produced after C13-C14 double bond cleavage is indicative of the OOH-group at C15 position of the ketone group formed after loss of water molecule from hydroperoxy-arachidonic acid. Fragments attributed to polar head of PE with m/z 140.0 was also formed during MS2 fragmentation. Based on fragmentation pattern the species with m/z 798.531 was identified as 15-HpETE-PE.

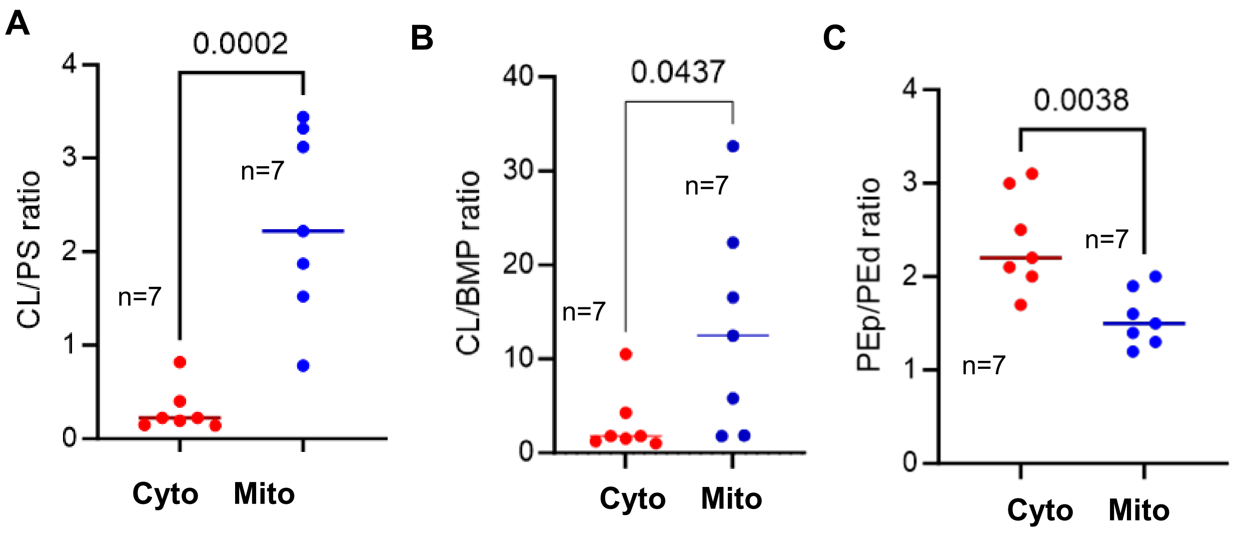

Supplementary Fig. 8. Phospholipid validation of compartmental purity

A) Highly significant differences and elevated CL/PS ratios and **B)** CL/BMP ratios in the mitochondrial and cytosolic fractions. **C)** Highly significant differences and elevated PEp/PEd ratios in the cytosol vs mitochondria by paired t-testing on n of 7 biologic replicates. Source data are provided as Source Data file.
