# Supplementary material for: Compartmentalized mitochondrial ferroptosis converges with optineurin-mediated mitophagy to impact airway epithelial cell phenotypes and asthma outcomes
Source: Nat Commun. 2024 Jul 10;15:5818. doi: 10.1038/s41467-024-50222-2 (PMC11237105; doi:10.1038/s41467-024-50222-2)
Supplement: Supplementary file 3 — Reporting Summary [file 41467_2024_50222_MOESM3_ESM.pdf]

nature portfolio

Corresponding author(s): Sally Wenzel MD and Jinming Zhao PhD

Last updated by author(s): 2024 06 11

Reporting Summary

Nature Portfolio wishes to improve the reproducibility of the work that we publish. This form provides structure for consistency and transparency in reporting. For further information on Nature Portfolio policies, see our Editorial Policies and the Editorial Policy Checklist.

Statistics

For all statistical analyses, confirm that the following items are present in the figure legend, table legend, main text, or Methods section.

n/a	Confirmed	
☐	☒	The exact sample size (*n*) for each experimental group/condition, given as a discrete number and unit of measurement
☐	☐	A statement on whether measurements were taken from distinct samples or whether the same sample was measured repeatedly
☐	☒	The statistical test(s) used AND whether they are one- or two-sided<br>*Only common tests should be described solely by name; describe more complex techniques in the Methods section.*
☒	☐	A description of all covariates tested
☒	☐	A description of any assumptions or corrections, such as tests of normality and adjustment for multiple comparisons
☐	☒	A full description of the statistical parameters including central tendency (e.g. means) or other basic estimates (e.g. regression coefficient) AND variation (e.g. standard deviation) or associated estimates of uncertainty (e.g. confidence intervals)
☐	☒	For null hypothesis testing, the test statistic (e.g. *F*, *t*, *r*) with confidence intervals, effect sizes, degrees of freedom and *P* value noted<br>*Give P values as exact values whenever suitable.*
☒	☐	For Bayesian analysis, information on the choice of priors and Markov chain Monte Carlo settings
☒	☐	For hierarchical and complex designs, identification of the appropriate level for tests and full reporting of outcomes
☒	☐	Estimates of effect sizes (e.g. Cohen's *d*, Pearson's *r*), indicating how they were calculated

Our web collection on statistics for biologists contains articles on many of the points above.

Software and code

Policy information about availability of computer code

Data collection	There were no computer codes used in the analyses related to this manuscript
Data analysis	Data analysis was all done using JJ GraphPad Prism software version 7

For manuscripts utilizing custom algorithms or software that are central to the research but not yet described in published literature, software must be made available to editors and reviewers. We strongly encourage code deposition in a community repository (e.g. GitHub). See the Nature Portfolio guidelines for submitting code & software for further information.

Data

Policy information about availability of data

All manuscripts must include a data availability statement. This statement should provide the following information, where applicable:

- Accession codes, unique identifiers, or web links for publicly available datasets
- A description of any restrictions on data availability
- For clinical datasets or third party data, please ensure that the statement adheres to our policy

De-identified data from both in vitro and ex vivo samples from human participants will be available on request.

Research involving human participants, their data, or biological material

Policy information about studies with human participants or human data. See also policy information about sex, gender (identity/presentation), and sexual orientation and race, ethnicity and racism.

Reporting on sex and gender	For in vitro experiments, typically with 3-6 samples from individual human participants, neither the biologic sex nor the societal gender were reported. For those sample sizes, seeing differences would be extraordinary. Please see Table 1 and 2 which includes biologic sex related to the ex vivo studies.
Reporting on race, ethnicity, or other socially relevant groupings	We report race as Black, White or other in Table 2 (for the ex vivo study of ciliated cell phenotype). As is apparent only one participant was enrolled as "other"
Population characteristics	We report asthma as severe or mild-moderate. These definitions are based on the European Respiratory Society and American Thoracic Society definitions of severe asthma from 2013 (Chung, K.F., Wenzel, S. & European Respiratory Society/ American Thoracic Society Severe Asthma International Guidelines Task, F. From the authors: International European Respiratory Society/American Thoracic Society guidelines on severe asthma. Eur Respir J 44, 1378-1379 (2014). This definition is based on clinical symptoms (asthma control), exacerbations, lung function changes and amount of medications used. Lung function (FEV1) data are also included in the figures. Healthy individuals were those without any evidence of respiratory disease (or other chronic disease) and normal lung function.
Recruitment	Participants were recruited through the University of Pittsburgh Clinical and Translational Science Institute registry, the University of Pittsburgh Asthma and Environmental Lung Health registry, as well as through the pulmonary clinics. Social media ads (Facebook) were occasionally utilized.
Ethics oversight	University of Pittsburgh Institutional Review Board

Note that full information on the approval of the study protocol must also be provided in the manuscript.

Field-specific reporting

Please select the one below that is the best fit for your research. If you are not sure, read the appropriate sections before making your selection.

☒ Life sciences ☐ Behavioural & social sciences ☐ Ecological, evolutionary & environmental sciences

For a reference copy of the document with all sections, see nature.com/documents/nr-reporting-summary-flat.pdf

Life sciences study design

All studies must disclose on these points even when the disclosure is negative.

Sample size	For in vitro experiments, typically 3-6 samples from individual human participants were evaluated. This is relatively standard for in vitro studies and no sample size calculations were performed. The ex vivo studies of specific proteins of interest (by immunofluorescence) in freshly brushed human airway epithelial cells, sample size was limited by availability of bronchoscopic specimens, and also not by sample size calculations. For the ex vivo studies comparing 15LO1 protein expression with ciliated cell phenotype, a pilot analysis supported that using a standard deviation of 7-8% for ciliated cell percentages, a sample size of 35-40 participants would be sufficient with 90% power and an alpha of 0.05.
Data exclusions	No data were excluded.
Replication	The ex vivo studies of ciliary length were not replicated using another center's data. This would be very difficult as few centers do bronchoscopies on asthmatic participants and very few collect samples for western blot/protein analysis.
Randomization	This was not a clinical trial, so there was no randomization
Blinding	This was not a clinical trial so there was no blinding

Reporting for specific materials, systems and methods

We require information from authors about some types of materials, experimental systems and methods used in many studies. Here, indicate whether each material, system or method listed is relevant to your study. If you are not sure if a list item applies to your research, read the appropriate section before selecting a response.

Materials & experimental systems

n/a	Involved in the study
☐	☒ Antibodies
☒	☐ Eukaryotic cell lines
☒	☐ Palaeontology and archaeology
☒	☐ Animals and other organisms
☐	☒ Clinical data
☒	☐ Dual use research of concern
☒	☐ Plants

Methods

n/a	Involved in the study
☒	☐ ChIP-seq
☒	☐ Flow cytometry
☒	☐ MRI-based neuroimaging

Antibodies

Antibodies used	Antibodies against LC3 (rabbit IgG) was purchased from Sigma-Aldrich (St. Louis, MO). 15LO1 (rabbit IgG) was from Abnova (Walnut, CA). PINK1 (rabbit IgG), LC3B (rabbit IgG) and GAPDH (goat IgG) was from NOVUS (Littleton, CO). pParkin (rabbit IgG) and OPTN (rabbit IgG) was from Proteintech (Rosemont, IL) PEBP1 (mouse IgG) and OPTN (mouse IgG) was from Santa Cruz (Santa Cruz, CA). GPX4 (rabbit IgG), MTCO2 (mouse IgG) and Total OXHPOS (mouse IgG) was from Abcam (Cambridge, MA). ATP synthase (Mouse IgG) was from Invtrogen (Carlsbad, CA). The anti-GAPDH antibody was from Novus Biologicals (Littleton, CO).
Validation	Most of the antibodies have been validated and applied in our previous publications. PINK1, pPARKIN, OPTN and ATP synthase have been validated by manufacturers with multiple publication references.

Clinical data

Policy information about clinical studies

All manuscripts should comply with the ICMJE guidelines for publication of clinical research and a completed CONSORT checklist must be included with all submissions.

Clinical trial registration	Since this is not a clinical trial, there is no registration
Study protocol	The study protocol can be provided on request. This is an observational study which focuses on collection of lung-specific biologic samples (Immune Mechanisms of Severe Asthma/NIAID and Protein-Oxidized Phospholipid Interactions Determine Epithelial Cell Fate and Asthma Control/NIAID)
Data collection	Samples were all collected as part of IRB approved research bronchoscopies with associated clinical visits where asthma control questionnaires, spirometry and asthma related clinical history was obtained.
Outcomes	As this is an exploratory study of mechanisms of severe asthma, there is no primary outcome
