# Supplementary material for: Compartmentalized mitochondrial ferroptosis converges with optineurin-mediated mitophagy to impact airway epithelial cell phenotypes and asthma outcomes
Source: Nat Commun. 2024 Jul 10;15:5818. doi: 10.1038/s41467-024-50222-2 (PMC11237105; doi:10.1038/s41467-024-50222-2)
Supplement: Supplementary file 4 — Source Data [file 41467_2024_50222_MOESM4_ESM.zip › Raw WB image-Fl Nat Com 2024.pdf]

Fig. 2. Mitochondrial 15LO1 pathway activity drives compartmentalized ferroptosis.

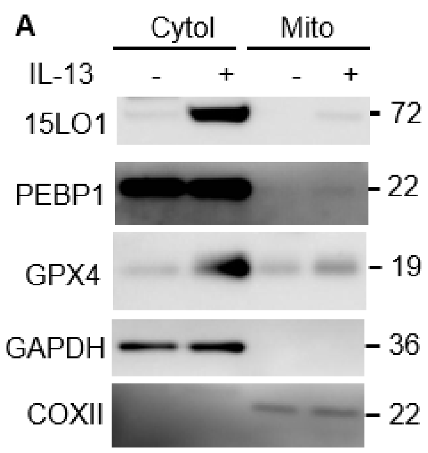

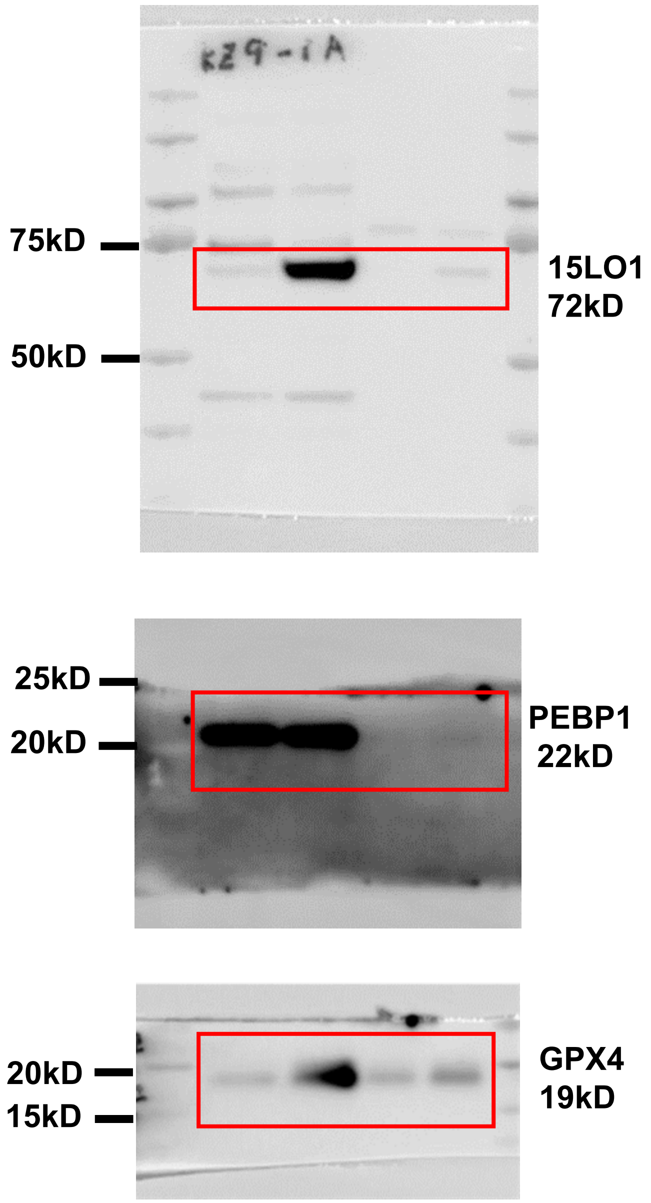

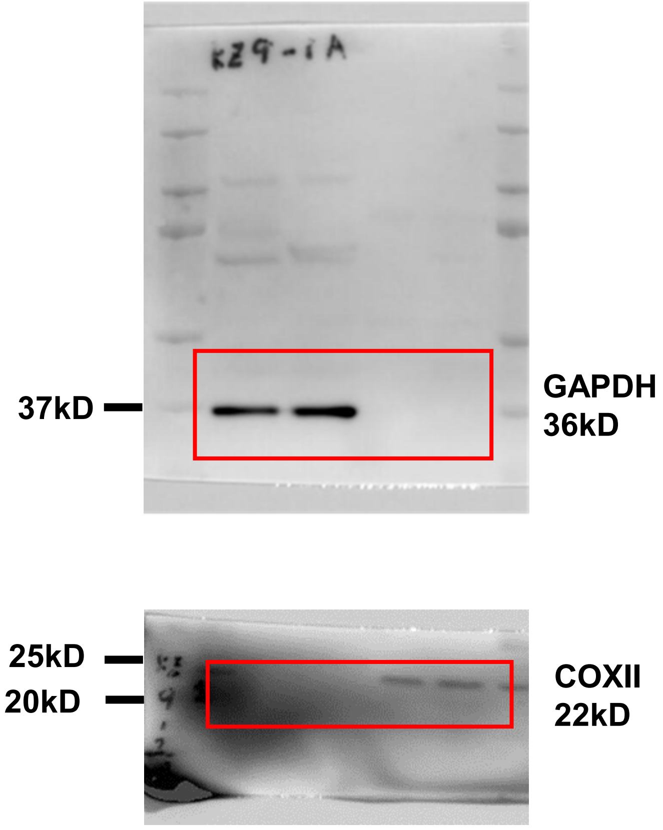

ALOX15	Abnova	#H00000246-D01P
RKIP(H-10)	Santa Cruz	#sc-376925
Glutathione Peroxidase	Abcam	#ab125066
GAPDH	NOVUS	#NB300-320
MTCO2	Abcam	#ab110258

Fig. 4A

A) IL-13-induces 15LO1 and LC3-II expression by WB over time, and 15LO1 increases prior to LC3-II.

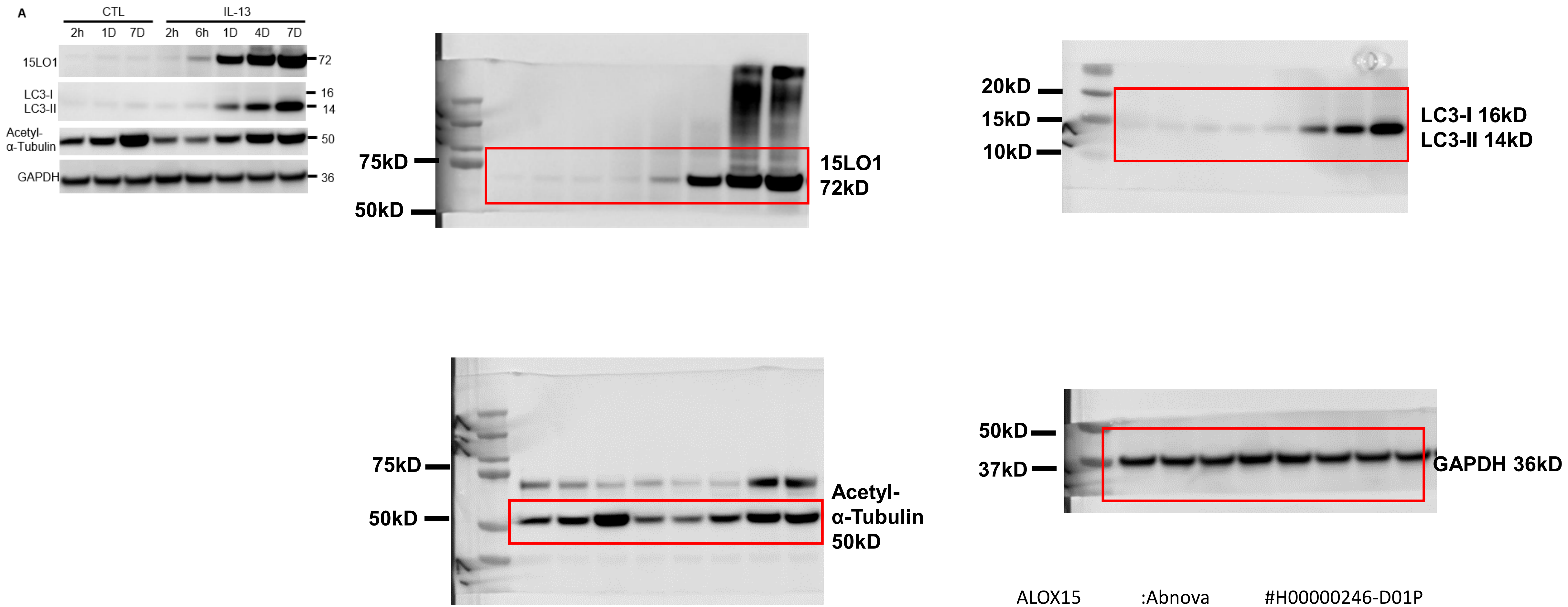

ALOX15	:Abnova	#H00000246-D01P
LC3B antibody	:Sigma	#L7543
Acetyl-alfa tubulin:	Sigma	#T6793
GAPDH	:NOVUS	#NB300-320

Fig. 4D IL-13 induces LC3 expression with enrichment of activated LC3-II in mitochondrial fractions

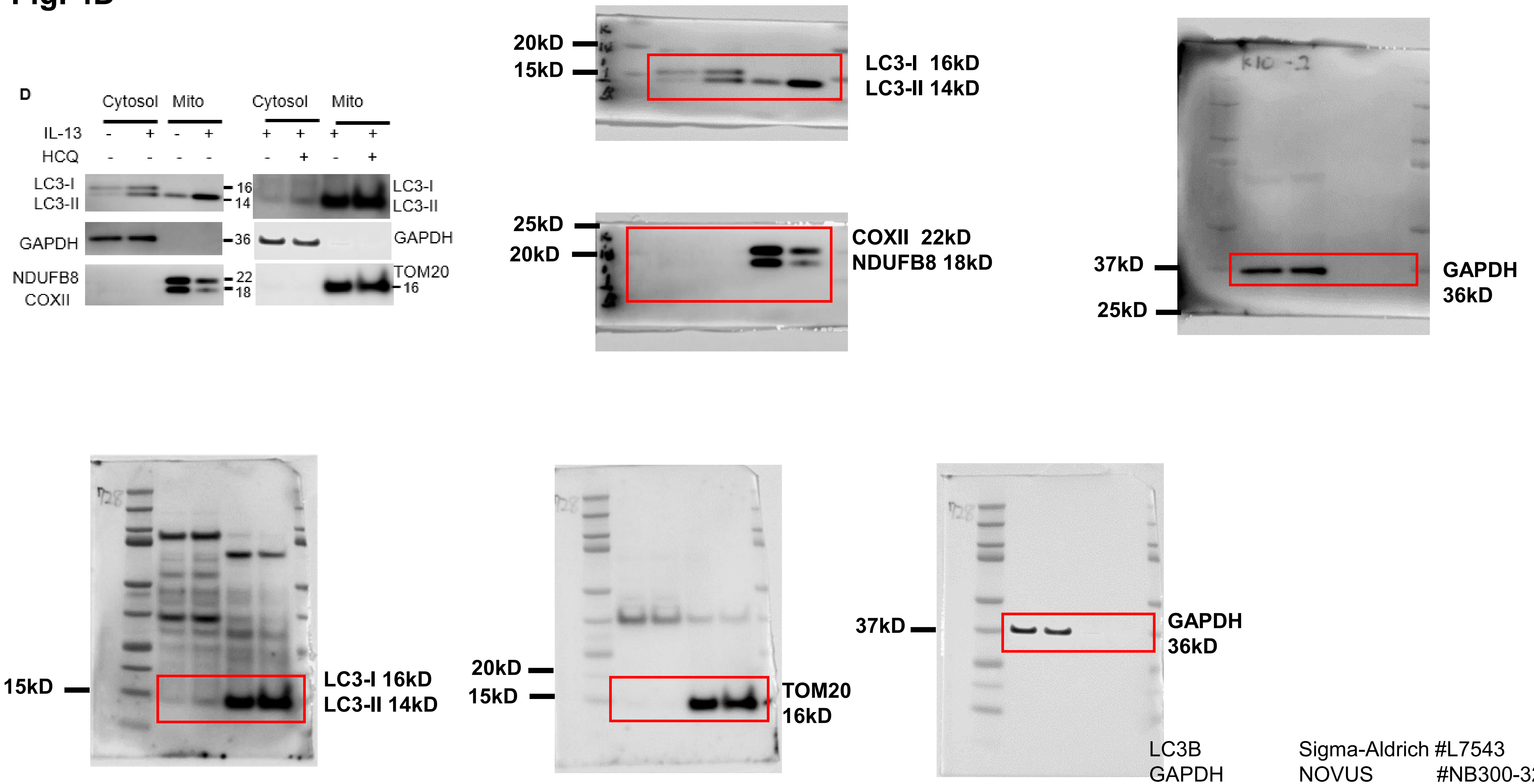

LC3B	Sigma-Aldrich	#L7543
GAPDH	NOVUS	#NB300-320
Total OXPHOS	Abcam	#ab110411
TOM20	Santa Cruz	# sc-11415

Fig. 4G 15LO1 KD (siALOX15) lowered mitochondrial LC3-II under IL-13 conditions.

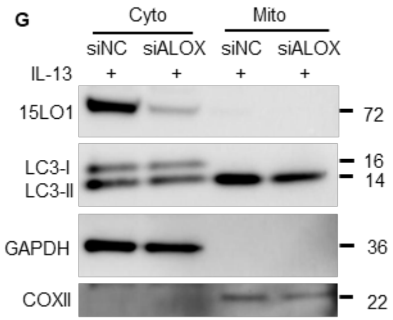

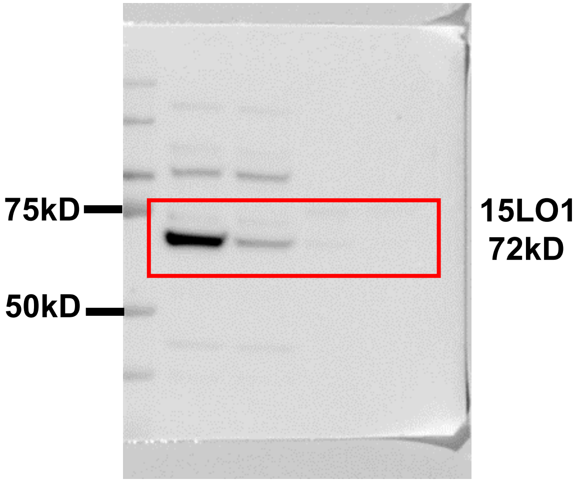

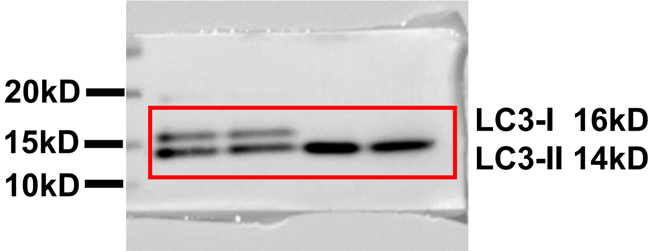

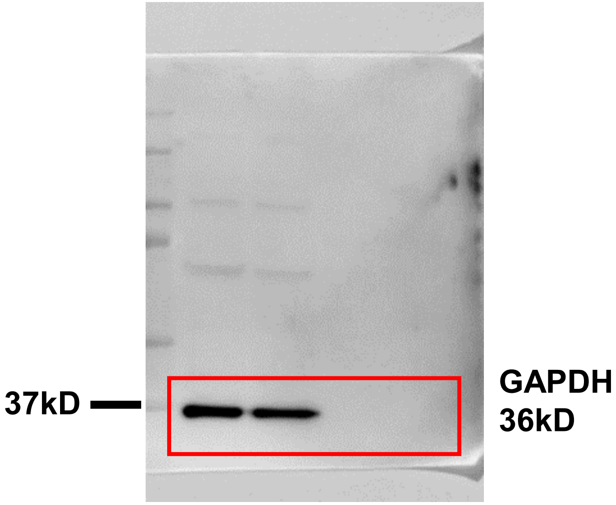

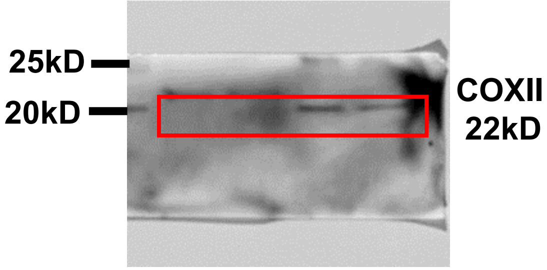

ALOX15	Abnova	#H00000246-D01P
LC3B	Sigma-Aldrich #L7543	
GAPDH	NOVUS	#NB300-320
MTCO2	Abcam	#ab110258

Fig. 5A PINK1/pParkin/Optineurin (OPTN) /LC3-II are increased under IL13 conditions using Western Blot analysis

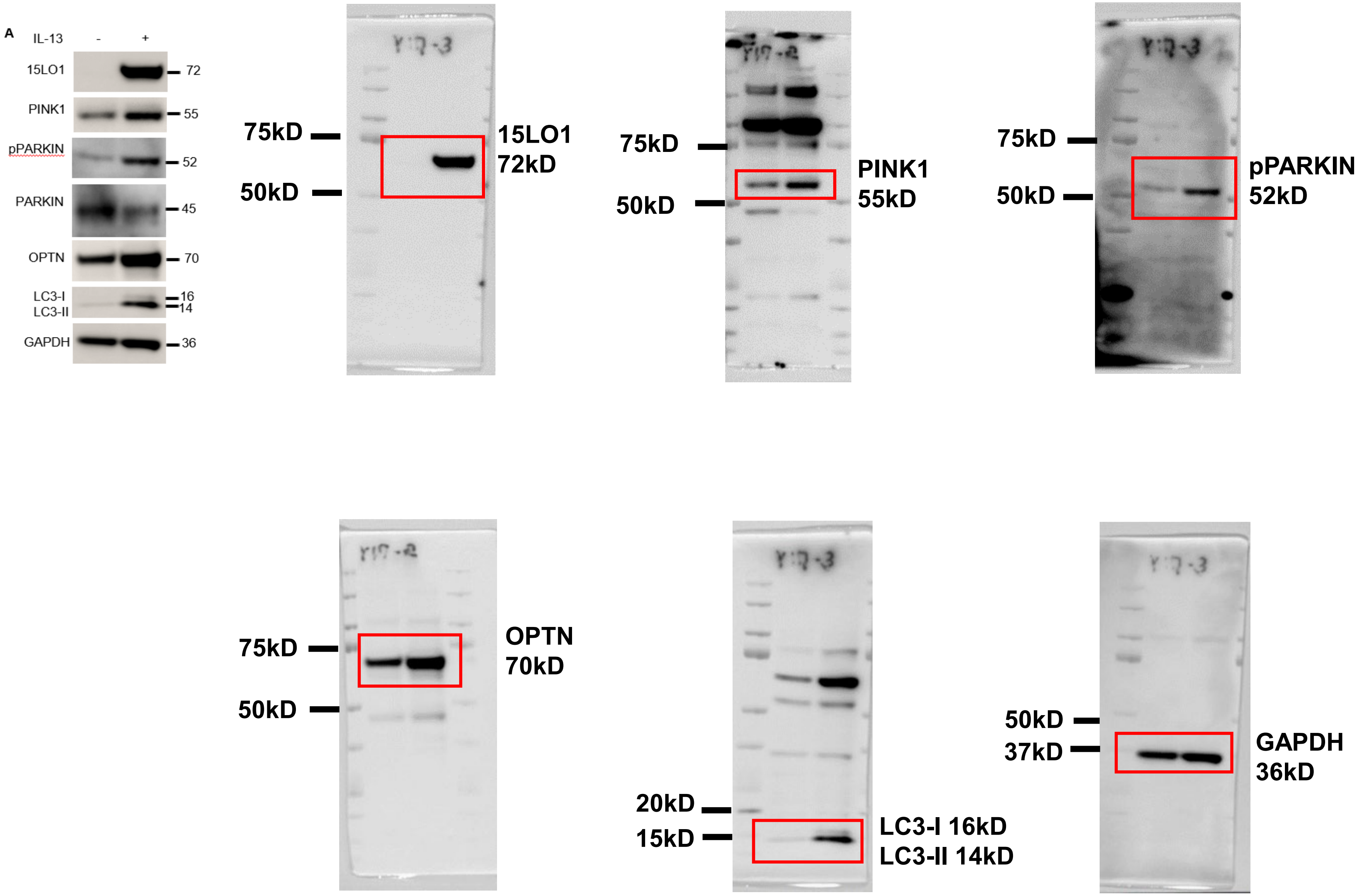

ALOX15	Abnova	#H00000246-D01P
PINK1	Santa Cruz	#sc-517353
pPARKIN	Affinity	#AFF-AF3500,
Parkin	Santa Cruz	#sc-32282
OPTN	Proteintech	#10837-1-AP
LC3B	Sigma-Aldrich	#L7543
GAPDH	NOVUS	#NB300-320

Fig. 5B IL-13 increases OPTN co-immunoprecipitation with LC3 (Co-IP/WB)

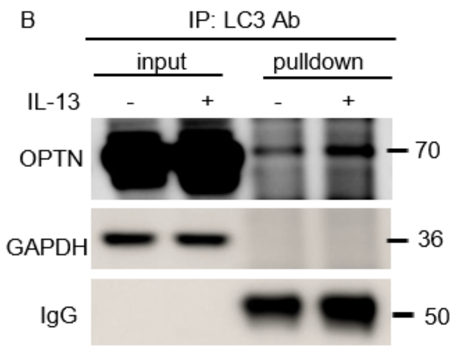

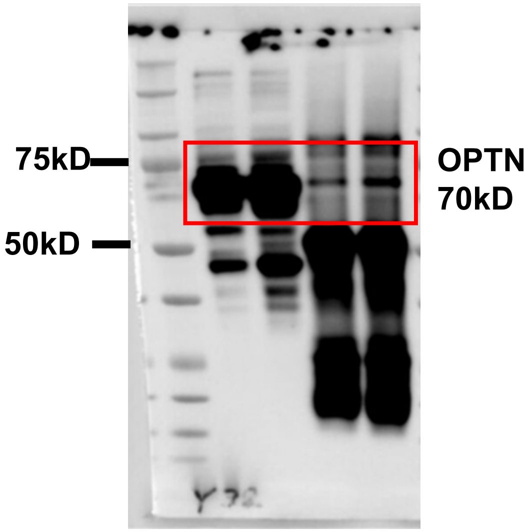

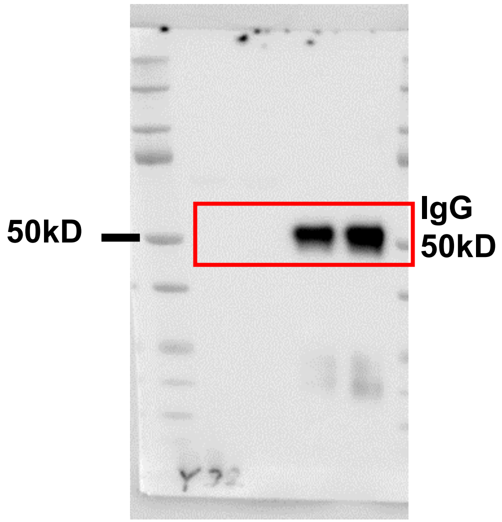

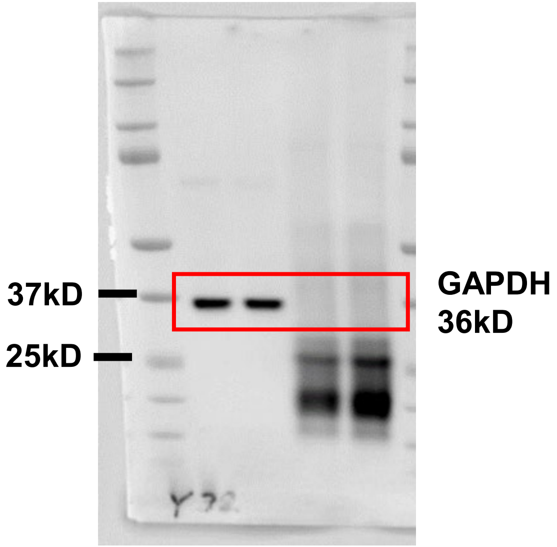

OPTN	Proteintech	#10837-1-AP
GAPDH	NOVUS	#NB300-320
IP:LC3B	NOVUS	#NB100-2220

Fig. 5D **D)** Pretreatment with FER-1 and/or BLX2477 suppress IL-13-induced LC3-II ALI for 9 days and IL-13 for 5 days, with/without simultaneous FER-1 (1μM) and/or BLX2477 (2μM) each for days

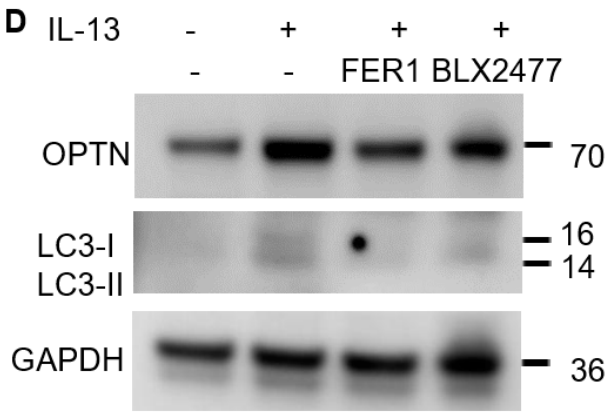

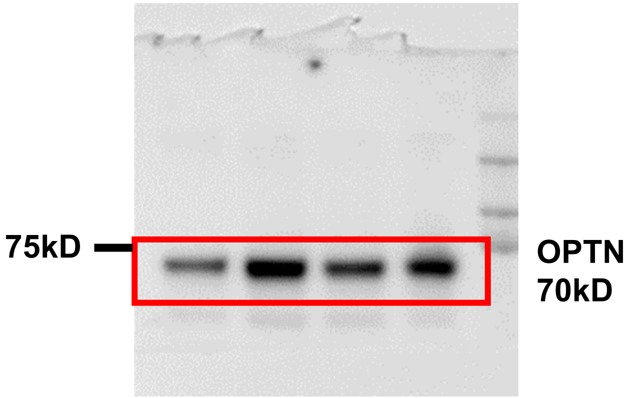

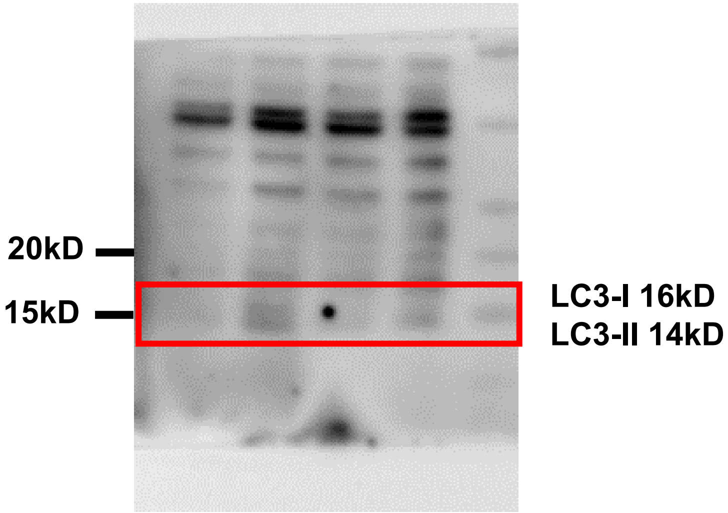

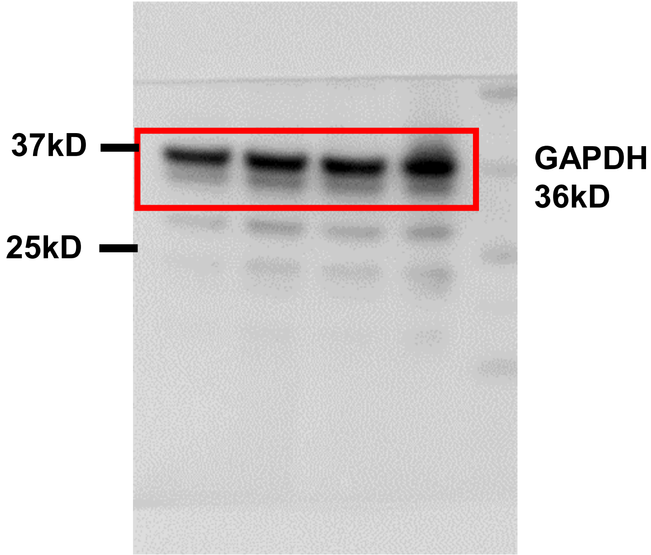

OPTN	Proteintech	#10837-1-AP
LC3B	Sigma-Aldrich	#L7543
GAPDH	NOVUS	#NB300-320

Fig. 5F 15LO1 KD/siLALOX15 lowers PINK1/PARKIN pathway activation (WB) and OPTN-LC3 binding (Co-PI/WB) under IL-13 conditions

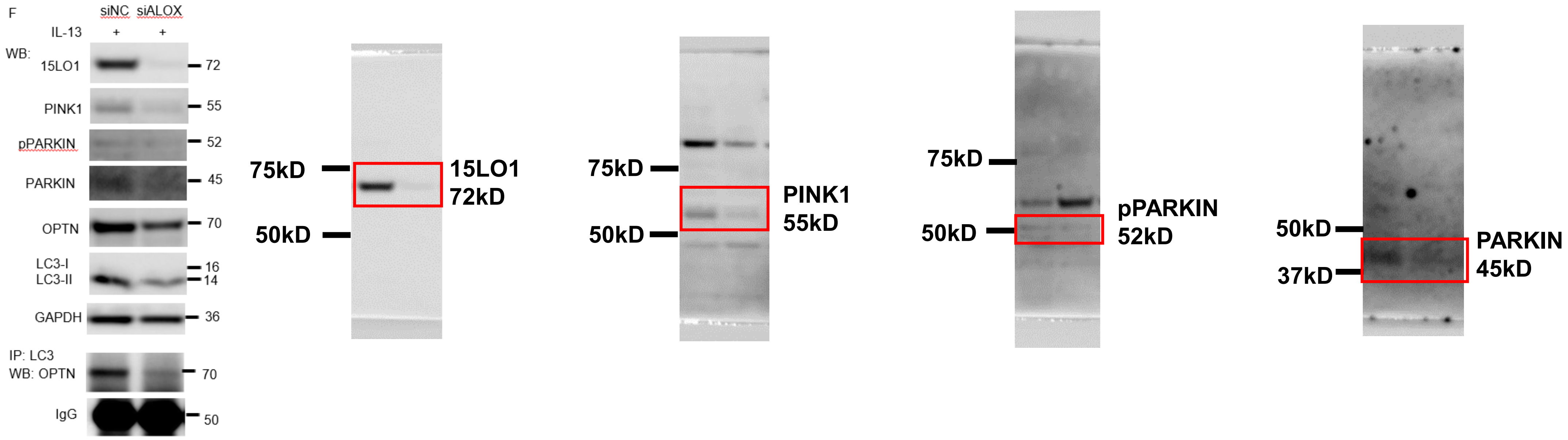

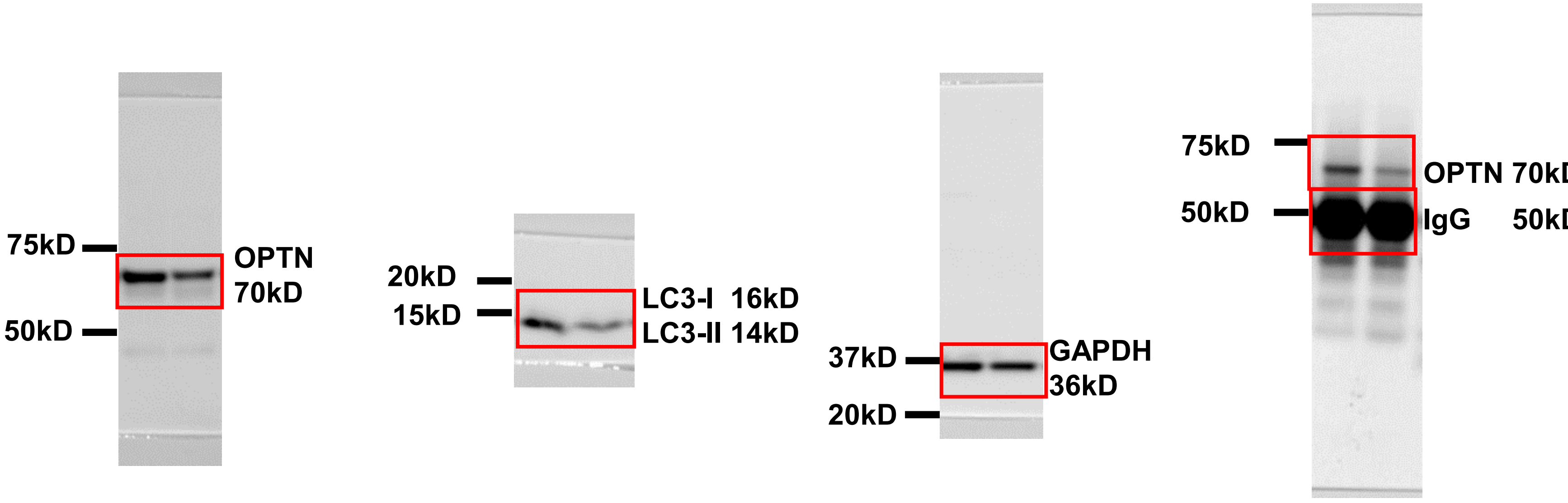

ALOX15	Abnova	#H00000246-D01P
PINK1	Santa Cruz	#sc-517353
pPARKIN	Affinity	#AFF-AF3500,
Parkin	Santa Cruz	#sc-32282
OPTN	Proteintech	#10837-1-AP
LC3B	Sigma-Aldrich	#L7543
GAPDH	NOVUS	#NB300-320
IP:LC3B	NOVUS	#NB100-2220

Fig. 5H

H) Higher OPTN expression in association with 15LO1 and LC3 in freshly brushed asthmatic HAECs as compared to healthy controls by WB

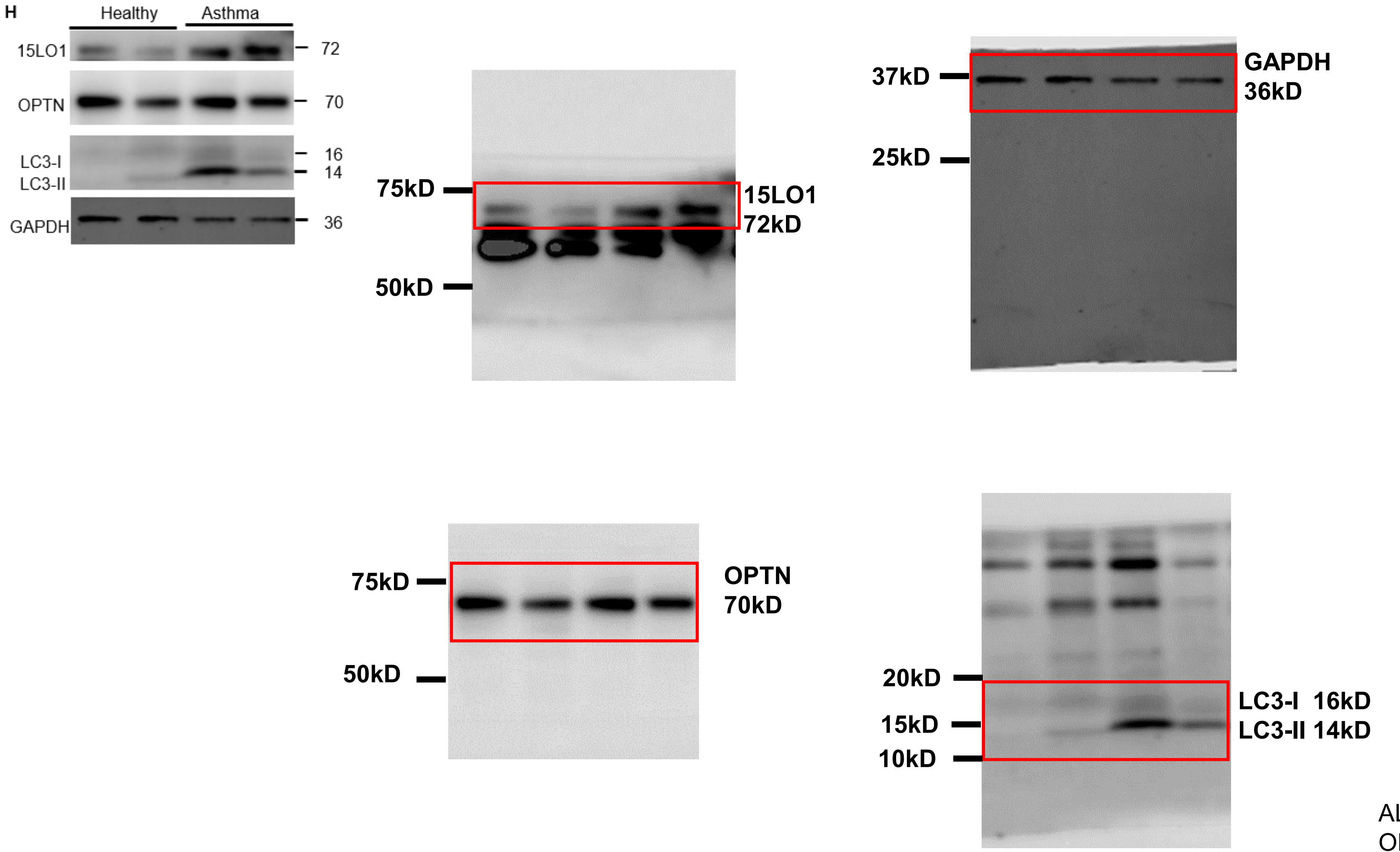

ALOX15	Abnova	#H00000246-D01P
OPTN	Proteintech	#10837-1-AP
LC3B	Sigma-Aldrich	#L7543
GAPDH	NOVUS	#NB300-320

Fig. 6D

D) Under the same conditions, FER-1 and BLX2477 increase TUB1A expression by WB under IL-13 conditions.

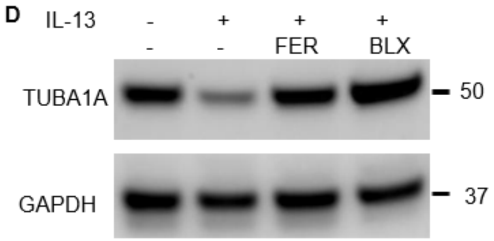

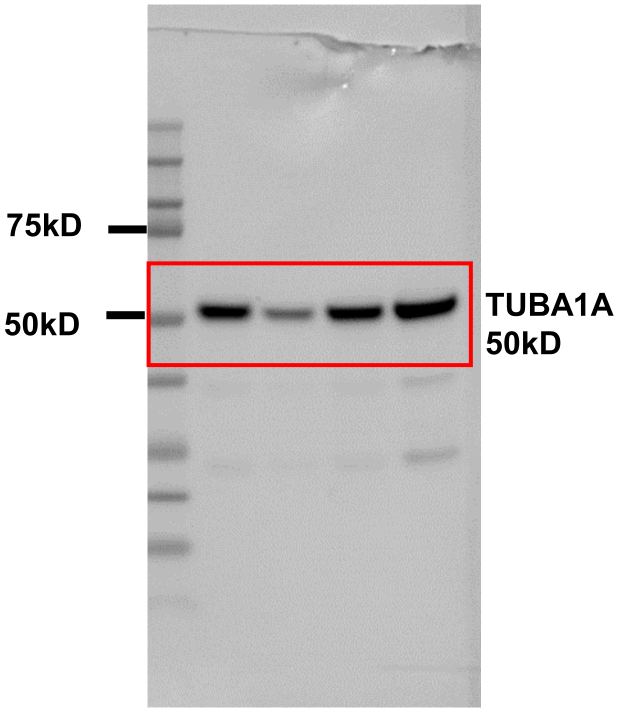

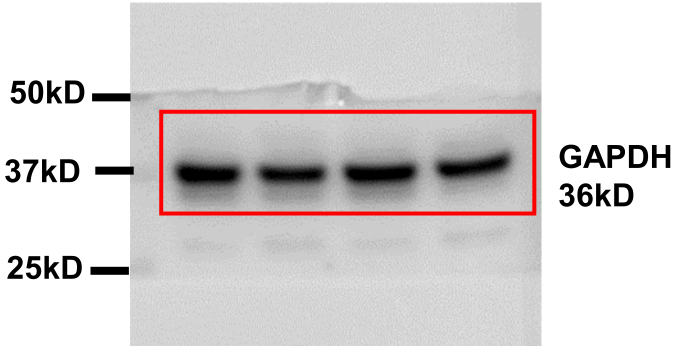

TUBA1A	Sigma	#T6793
GAPDH	NOVUS	#NB300-320

Fig. S1C **C)** IL-13 (10ng/ml, 7 days) does not induce cell death measured by nuclear DNA loss analysis (WB of Histidine)

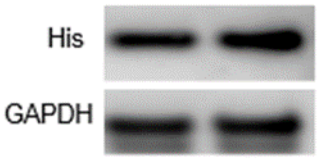

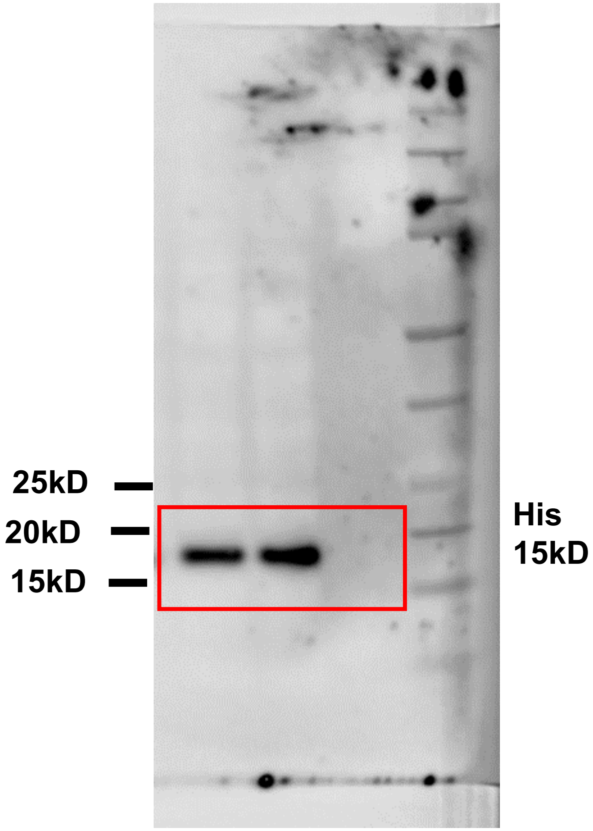

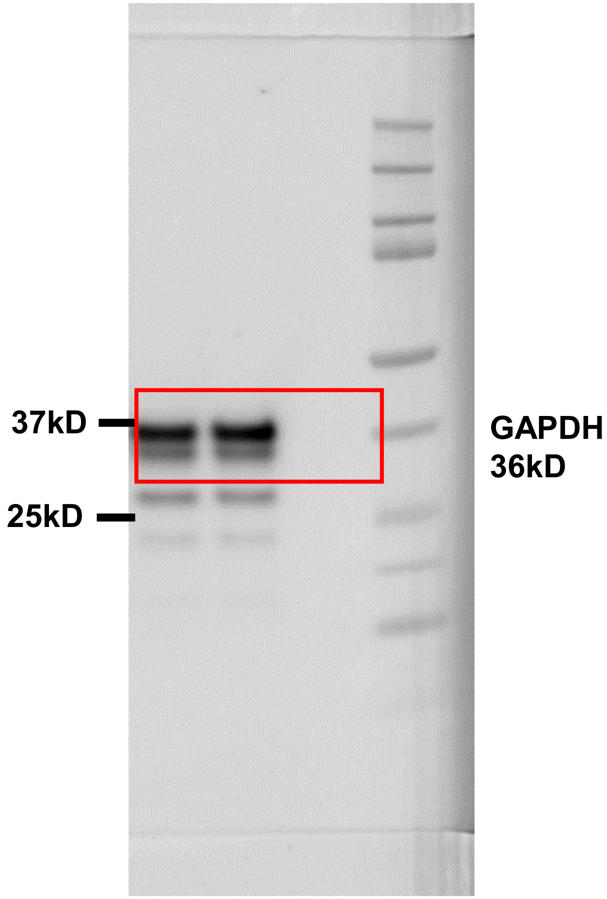

Histone Cell signaling technology, #971

GAPDH NOVUS #NB300-320

Fig. S5S

OPTN KD using siOPTN (see WB below)

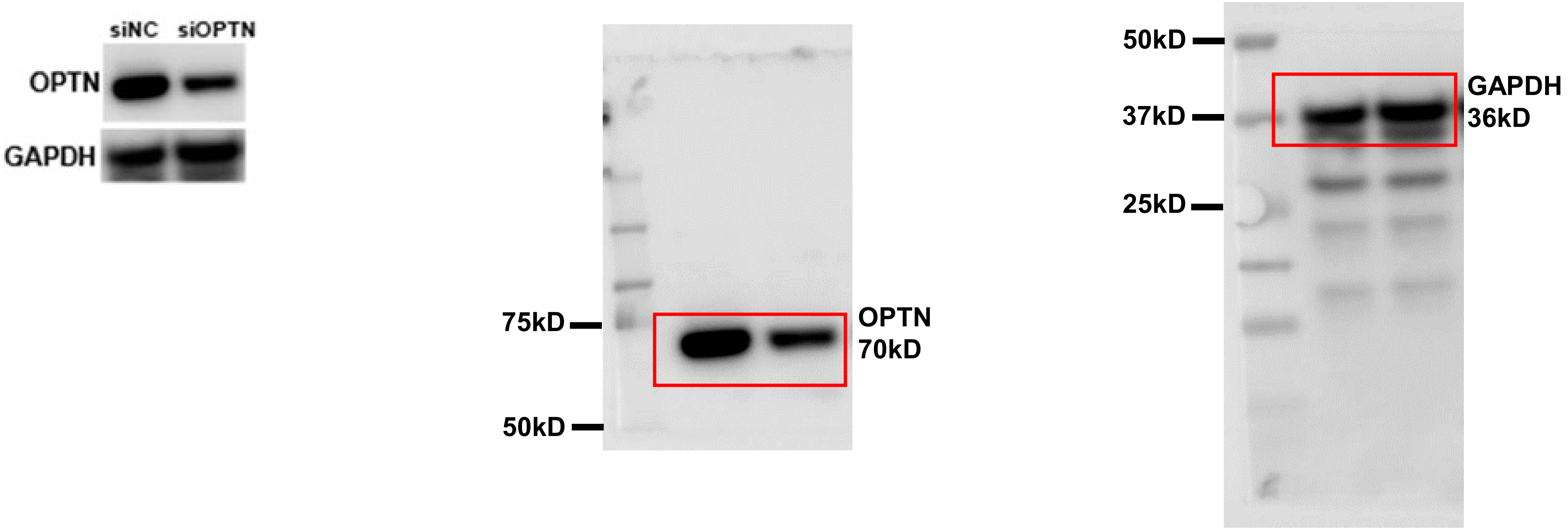

OPTN	Proteintech	#10837-1-AP
GAPDH	NOVUS	#NB300-320c
